# Investigating Flavonoids by HPTLC Analysis Using Aluminium Chloride as Derivatization Reagent

**DOI:** 10.3390/molecules29215161

**Published:** 2024-10-31

**Authors:** Sharmin Sultana, Md Lokman Hossain, Tom Sostaric, Lee Yong Lim, Kevin J. Foster, Cornelia Locher

**Affiliations:** 1 Division of Pharmacy, School of Allied Health, University of Western Australia, Perth 6009, Australia; sharmin.sultana@research.uwa.edu.au (S.S.); mdlokman.hossain@uwa.edu.au (M.L.H.); tom.sostaric@uwa.edu.au (T.S.); lee.lim@uwa.edu.au (L.Y.L.); 2 Institute for Pediatric Perioperative Excellence, The University of Western Australia, Perth 6009, Australia; 3 School of Agriculture and Environment, University of Western Australia, Crawley 6009, Australia; kevin.foster@uwa.edu.au; 4 Department of Primary Industries and Regional Department, Perth 6000, Australia

**Keywords:** flavonoids, derivatization, HPTLC, AlCl_3_, NaNO_2_-AlCl_3_-NaOH, spectral shift

## Abstract

This is the first study to report on high performance thin layer chromatography (HPTLC) generated spectrophotometric data to systematically capture flavonoid compounds using optimized derivatization with either AlCl_3_ or NaNO_2_-AlCl_3_-NaOH as visualisation reagents. While the traditional AlCl_3_ colorimetric method using UV–Vis analysis provides valuable insights into the presence of flavonoids and allows derivation of the total flavonoid content (TFC) of a sample, HPTLC fingerprints obtained after spraying with AlCl_3_ or NaNO_2_-AlCl_3_-NaOH enable the visualization of the various flavonoids present in a sample based on their respective absorption shifts, thus complementing the traditional TFC assay. In this study, 40 different flavonoids representing different classes (flavonols, flavanolols, flavan-3-ol, flavones, flavanones, and isoflavonoids) were analysed. Upon derivatization with AlCl_3_ most of the investigated flavonoids recorded bathochromic shifts, yielding characteristic λ_max_ values between 370 and 420 nm, while spraying with NaNO_2_-AlCl_3_-NaOH triggered hyperchromic shifts, and thus an increase in absorbance intensity in flavonoids with particular substitution patterns. A few non-flavonoid components with structural similarities to flavonoids (e.g., rosmarinic acid, gallic acid, aspirin, salicylic acid) served as the negative control in this study to determine whether the derivatization reagents allowed exclusive detection of flavonoids. The method was then applied to the analysis of flavonoid containing supplements as well as red clover honey to demonstrate the method’s application in the analysis of natural products.

## 1. Introduction

As plant secondary metabolites with a polyphenolic structure, flavonoids constitute an important class of natural products which are widely found in fruits, vegetables, and certain beverages such as tea and wine. They are present in several parts of plants [1] as they serve a wide range of roles, including growth promotion and defence against invading organisms [2]. They also act as flower pigments in most Angiosperm families to attract pollinators [1,3].

Due to their potential health-promoting effects, which include anti-oxidative, anti-inflammatory, anti-mutagenic, and anti-carcinogenic properties, coupled with the capacity to modulate key cellular enzyme functions such as xanthine oxidase, cyclo-oxygenase, lipoxygenase, and phosphoinositide 3-kinase [4,5,6], flavonoids are included as active ingredients in a variety of nutraceutical, pharmaceutical, and cosmetic products. Many studies have reported the applications of flavonoids for various purposes [5,6,7,8,9]. For example, Dixon and Pasinetti reviewed plant flavonoids and isoflavonoids in detail and discussed their applications in agriculture and human neuroscience [7]. Similarly, Kumar and Pandey reviewed the protective roles of flavonoids against human diseases as well as their functions in plants [8].

**Figure 1:**
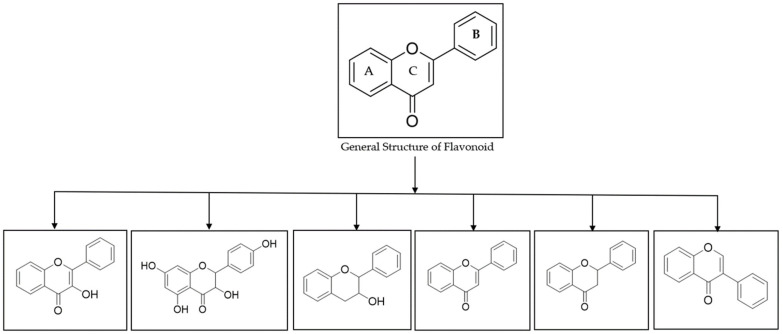
General structure and subclasses of flavonoids.

Chemically, flavonoids are based upon a fifteen-carbon skeleton consisting of two benzene rings (A and B as shown in Figure 1) linked via a heterocyclic pyran ring (C). Flavonoids can be subdivided into different subgroups such as flavonol, flavanonol, flavan-3-ol, flavones, flavanones, and isoflavones depending on the carbon of the C ring on which the B ring is attached to, the substitution pattern, degree of unsaturation, and oxidation of the C ring (Figure 1).

**Figure 2:**
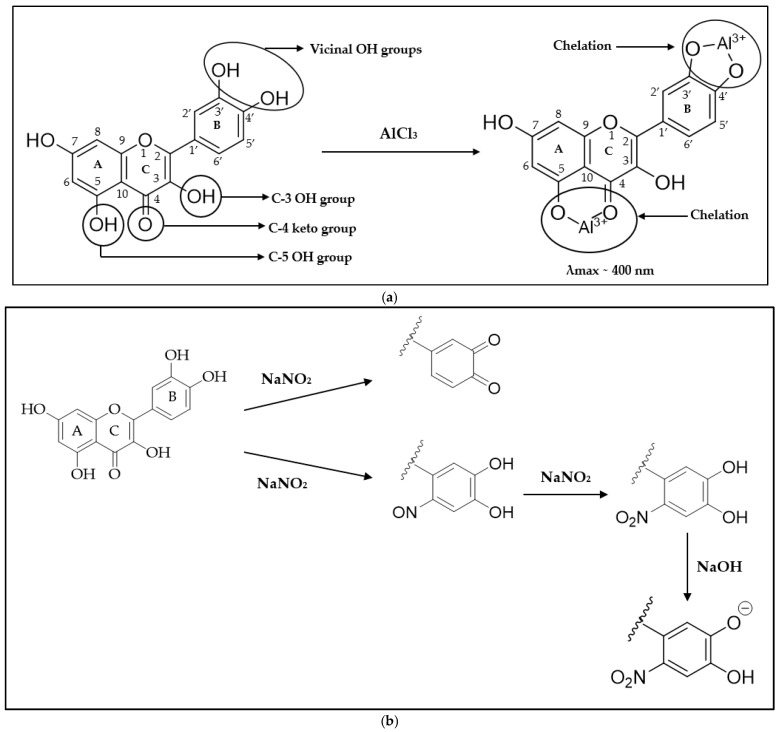
Complex formations of flavonoids: (**a**) Al (III)-flavonoid chelate; (**b**) potential reaction of flavonoids with NaNO_2_/NaOH.

An aluminium chloride (AlCl_3_) colorimetric method was first proposed by Christ and Müller in 1960 for the quantification of flavonol derivatives in drugs [9]. The chemical principle underpinning this method is the formation of an acid-stable complex involving the Al^3+^ cation, the C-4 keto group of the flavonoid and either its C-3 or C-5 hydroxyl group (Figure 2a). In addition, the AlCl_3_ reagent also forms acid labile complexes with the vicinal hydroxyl groups in the B ring of flavonoids (Figure 2a). The absorbance maximum of the Al (III)-flavonoid chelates was reported to be around 400 nm. Over time, this original colorimetric method underwent several modifications such as the introduction of sodium nitrite (NaNO_2_) and sodium hydroxide (NaOH) solutions alongside the AlCl_3_ reagent. The rationale for the introduction of these additional reagents was that sodium nitrite serves as an oxidation and nitrating agent selective for aromatic vicinal diols [10], thus yielding o-quinones and flavonoid–nitroxyl derivatives (Figure 2b). In a basic environment, the latter was reported to yield a distinct new absorbance maximum at approximately 500 nm on chelation with Al^3+^ [11]. Moreover, NaOH is utilized to create alkaline conditions that enhance the colour development reaction of flavonoids, contributing to the accurate determination of flavonoid content in various plant extracts [12].

While it is useful and thus popular to determine the total flavonoid content (TFC) of a sample using Al^3+^ based colorimetric methods, this analysis does not offer insights into the number and type of flavonoids contributing to the TFC reading. A preceding HPTLC-based chromatographic separation prior to derivatization with AlCl_3_ was therefore investigated in this study as a complementary analysis to the traditional TFC determination. The identification of flavonoids using derivatization with AlCl_3_ and NaNO_2_-AlCl_3_-NaOH following HPTLC analysis was inspired by HPTLC-DPPH analysis which enables the visualization of individual antioxidant components present in a sample, thus complementing the traditional DPPH-based antioxidant assay that captures the total antioxidant activity of the sample [13]. The aim was to develop first an optimized derivatization approach using either AlCl_3_ or a mixture of NaNO_2_-AlCl_3_-NaOH that facilitates the visualization of flavonoids but does not generate false positive results for non-flavonoid compounds and then to demonstrate the applicability of this optimized method for the analysis of some natural products.

## 2. Results

Based on the mechanism of the colorimetric method to determine TFC, the reaction sites for both derivatizing reagents, AlCl_3_ and NaNO_2_-AlCl_3_-NaOH, were categorized into three subclasses: chelation options with ring A and C for Al^3+^ (Figure 2a), chelation options with vicinal OH groups on ring B for Al^3+^, and also when using NaNO_2_-AlCl_3_-NaOH as derivatization reagent (Figure 2b). The number of potential chelation options for each flavonoid is shown in Section 2.1 and the Supplementary File.

**Figure 3:**
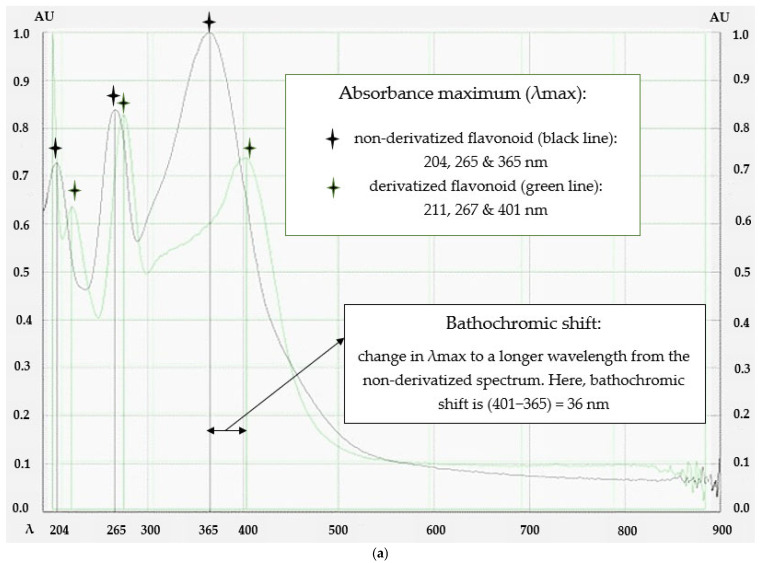

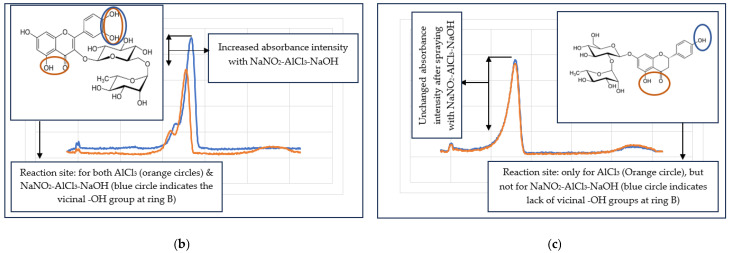
HPTLC-generated data: (**a**) absorbance maxima (λ_max_) of rutin before derivatization with AlCl_3_, and absorbance maxima and bathochromic shift of rutin after derivatization with AlCl_3_, (**b**) increase in absorbance intensity (hyperchromic shift) of rutin with the derivatizing reagents; (**c**) no change in absorbance intensity for naringin upon derivatization with NaNO_2_-AlCl_3_-NaOH.

After the application of 2% AlCl_3_, the absorbance maximum for most of the analysed flavonoids (Section 4.1) was found to be around 400 nm which was the result of a bathochromic shift, thus an increase in the absorbance maximum λ_max_, of approximately 30 to 100 nm compared to the λ_max_ of the non-derivatized flavonoid (Figure 3a) depending on the respective flavonoid subclass (Section 2.1). A similar absorbance maximum in the derivatized sample was also found on the application of 2% NaNO_2_-AlCl_3_-NaOH. However, an additional hyperchromic shift (a higher absorbance measured for the same concentration of analyte), thus an increase in absorbance intensity (defined in this study as >5%), could be recorded for those samples that feature the chelation option with vicinal OH groups at ring B (Figure 3b) and thus were able to form the flavonoid–nitroxyl chelate. On the other hand, absorbance intensity remained unchanged for all the analysed flavonoids given their lack of vicinal OH groups in ring B (Figure 3c). The investigated non-flavonoid standards did not conform to this pattern, producing much smaller bathochromic shifts of approximately 5 to 20 nm and yielding different λ_max_ values upon derivatization (Section 2.2). This confirms that the developed method is suitable to reliably detect flavonoid compounds without producing false positives (Section 2.3).

### 2.1. Flavonoids

Forty different standards representing different flavonoid subclasses were analysed in this study to observe potential bathochromic shifts and the effect on absorbance intensities after derivatization with both optimized reagents. Table 1 summarizes the findings for some of these flavonoids to illustrate the observed trends for each flavonoid subclass; individual data for all investigated flavonoids are included in the Supplementary Materials.

### 2.2. Non-Flavonoids

**Figure 4:**
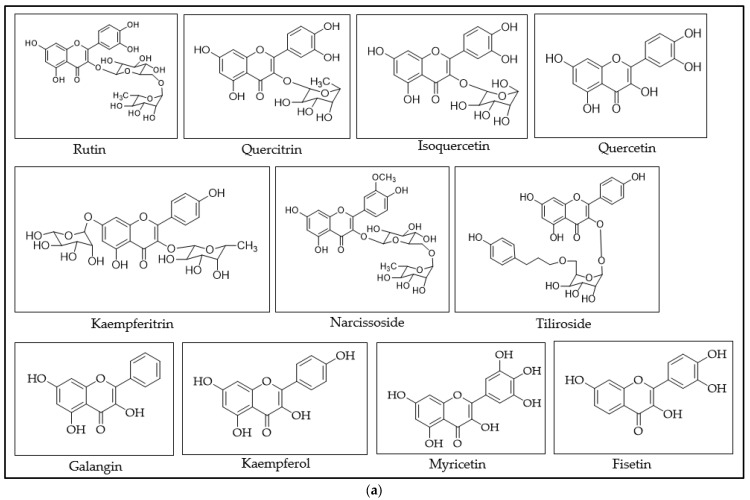

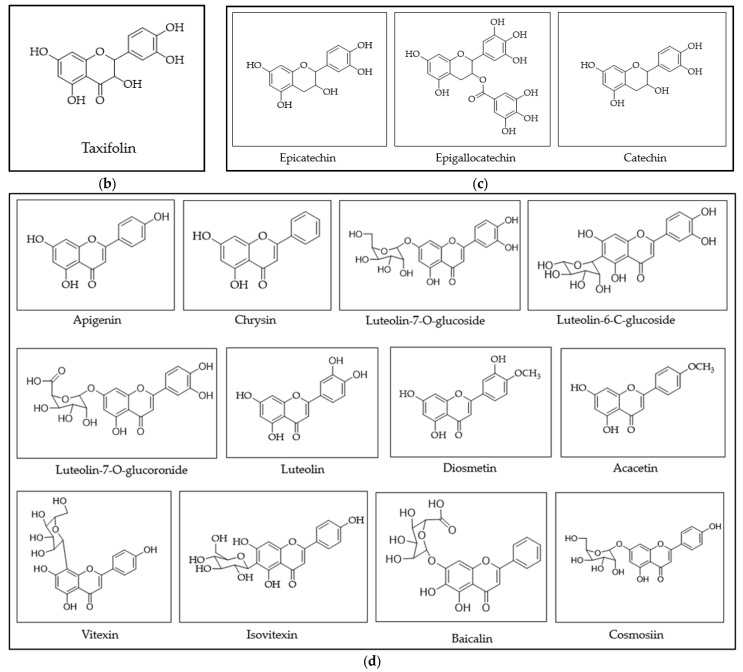

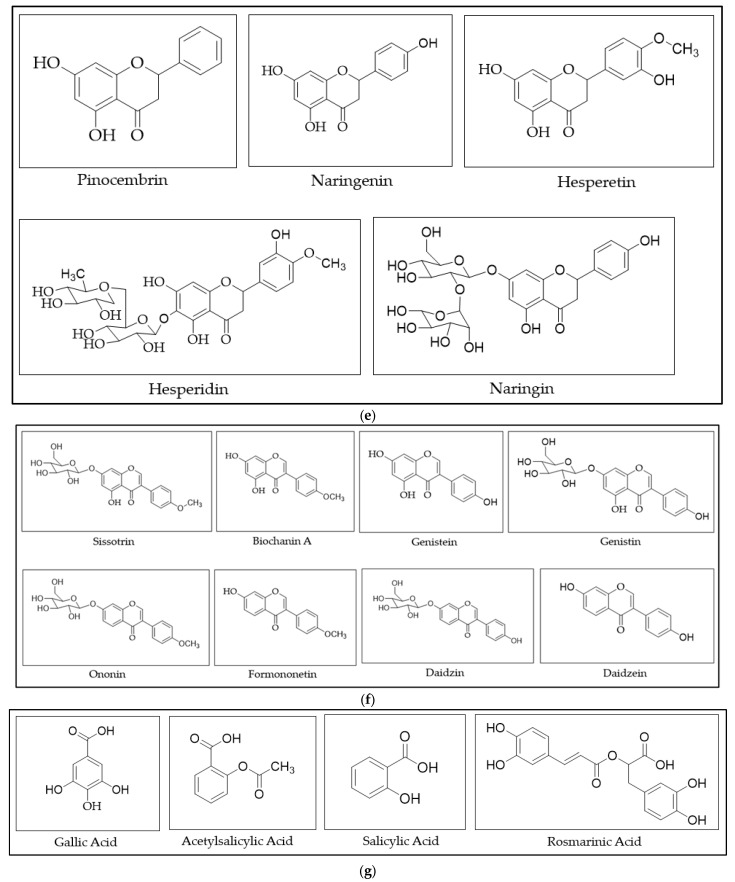
Chemical structures of flavonoid and non-flavonoid standards; (**a**): flavonols, (**b**): flavanolol, (**c**): flavan-3-ol, (**d**): flavones, (**e**): flavanones, (**f**): isoflavones and (**g**): non-flavonoids.

With λ_max_ values between 282 and 350 nm on derivatization with AlCl_3_ the four investigated non-flavonoids presented absorbance maxima that did not fall into the λ_max_ range typically observed for the investigated flavonoids. They also recorded a bathochromic shift of less than 20 nm from the non-derivatized spectrum (Table 2). Furthermore, on derivatization with NaNO_2_-AlCl_3_-NaOH, the previously recorded peak maxima and absorbance intensities remained unchanged (Figure 2b, Figure 3 and Figure 4g).

### 2.3. Flavonoid Identification in Some Natural Products

To demonstrate the application of the optimized derivatization method for the analysis of natural products, it was applied to two nutraceutical formulations containing either rutin or naringin as well as to a red clover honey extract. Methanolic solutions of rutin and naringin capsules (50 μg/mL) showed an absorbance maximum at 402 and 386 nm, respectively, after derivatization with AlCl_3_ (Table 3), which constituted a bathochromic shift of 38 and 99 nm in comparison with their respective spectra prior to derivatization. On derivatization with NaNO_2_-AlCl_3_-NaOH, the previously recorded peak maxima remained unchanged, but an increase in absorbance intensity could be noted for the rutin supplement only, which is consistent with observations made with rutin and naringin standards (Table 1) and is also in line with the structural requirements necessary for this increase in absorbance intensity. One of the bands in the investigated red clover honey extract presented a peak maximum at 370 nm after derivatization with AlCl_3_, translating into a bathochromic shift of 67 nm from the non-derivatized spectrum (Table 3). On derivatization with NaNO_2_-AlCl_3_-NaOH, the absorbance intensity remained unchanged for this band, which is consistent with the patterns established in this study for different flavonoid subclasses as this particular band in red clover honey had previously been identified as genistein [14] which is an isoflavonoid compound with a chelation option with Al^3+^ on rings A and C only (Figure 4f). Due to a lack of vicinal OH groups in ring B, the absorbance intensity remained unchanged on the application of NaNO_2_-AlCl_3_-NaOH.

## 3. Discussion

The qualitative identification of flavonoids using AlCl_3_ and NaNO_2_-AlCl_3_-NaOH as derivatization reagents following HPTLC analysis was inspired by HPTLC-DPPH analysis which enables the visualization of individual antioxidant components present in a sample and thus complements the traditional DPPH assay to capture total antioxidant activity [13,15,16]. The hypothesis of this study was that HPTLC analysis coupled with derivatization using AlCl_3_ and also NaNO_2_-AlCl_3_-NaOH might be able to detect individual flavonoid compounds based on specific absorption patterns (λ_max_) for each type of flavonoid, thus providing valuable insights into the diversity of flavonoids present in a sample, rather than purely capturing the total flavonoid content in that sample that is normally determined in the commonly employed AlCl_3_ colorimetric method.

Determination of total flavonoid content by colorimetry using AlCl_3_ with or without NaNO_2_ and NaOH is a very widely used method [11,12]. As part of this study to determine the optimal assay conditions, a comprehensive investigation was conducted considering different concentrations of the two reagents applied either as single or multiple sprays, the potential need for heating and the most suitable heating time, as well as the potential waiting time after the application of the derivatization reagent to allow for a complete reaction. A concentration of 2% AlCl_3_ applied in a single application without subsequent heating and with immediate visualization was found to be the most suitable and convenient approach. On the other hand, a mixture of 2% NaNO_2_-AlCl_3_-NaOH applied in a single spray without heating and followed by immediate analysis enabled the visualization of an increase in absorbance intensity in those flavonoids that presented a chelation option with vicinal OH groups in ring B and thus were able to form a flavonoid–nitroxyl chelate (Figure 2b, Figure 3 and Figure 4).

The study demonstrates that derivatizing with AlCl_3_ alone is sufficient to detect flavonoid compounds that present a suitable chelation option between rings A and C. This study was able to capture most flavonoids with the exception of some isoflavonoids that lack these structural arrangements (i.e., ononin, formononetin, daidzein, and daidzin—Figure 4f). The absorbance maximum for most of the analysed flavonoids on derivatization with 2% AlCl_3_ was found to be around 400 nm which complies with the principle of the colorimetric method established for the determination of TFC (Figure 2a). A bathochromic shift of between 30 and 105 nm from the non-derivatized spectrum was recorded depending on the flavonoid subclass. The investigated flavonols, flavanolol, flavan-3-ol, flavones, flavanones, and isoflavones presented bathochromic shifts resulting in newly formed λ_max_ values of 388–418, 390, 400, 380–389, 380–385, and 370–373 nm, respectively (Table 1 and Tables S1–S6 in Supplementary Materials). This new absorbance maximum around 400 nm of the flavonoid–aluminium complex is a unique characteristic that differentiates flavonoid compounds from other compounds, as was demonstrated with the investigation of non-flavonoid standards that served as negative control and did not comply with these spectral patterns.

Based on a review of the literature, this study also investigated the use of a combination of NaNO_2_ and NaOH with AlCl_3_ in the HPTLC-based colorimetric method. The aim was to employ sodium nitrite as a nitrating agent that selectively acts on aromatic vicinal diols [10] thus yielding flavonoid–nitroxyl chelates with a distinct new absorbance maximum at approximately 500 nm at basic pH. However, the λ_max_ of the investigated flavonoids recorded in this study on the application of 2% NaNO_2_-AlCl_3_-NaOH remained similar to their respective λ_max_ values that were recorded on the application of 2% AlCl_3_ only (approximately 400 nm), thus an additional bathochromic shift to around 500 nm as expected from descriptions in the literature could not be observed. However, a hyperchromic shift, thus an increase in absorbance intensity, on the formation of the flavonoid–nitroxyl chelate could be recorded for all flavonoids that feature the chelation option with vicinal OH groups in ring B. No change in absorbance intensity (<5%) was found for those flavonoids that did not present a suitable diol-substitution pattern. This means that AlCl_3_ and AlCl_3_-NaNO_2_-NaOH as derivatization reagents can be used not only to identify the presence of flavonoids (with the exception of some isoflavonoids) in a sample but can also reveal some information on specific structural features (i.e., presence or absence of vicinal aromatic diols—Figure 2b). The investigated non-flavonoids presented peak maxima (282–350 nm) distinctly different from the 400 nm determined as target λ_max_ for flavonoids (around 400 nm) on derivatization with AlCl_3_ and also presented negligible bathochromic shifts on exposure to the reagent (Table 2). Moreover, after derivatization with NaNO_2_-AlCl_3_-NaOH, the previously recorded peak maxima and absorbance intensities remained unchanged which confirms the suitability of the optimized method to reliably detect flavonoid compounds following HPTLC analysis. The study thus demonstrates that AlCl_3_ itself is sufficient as a derivatization reagent to qualitatively identify flavonoids present in a sample following HPTLC analysis (with the exception of some isoflavonoids). However, subsequently using NaNO_2_-AlCl_3_-NaOH as a second spray reagent on a newly developed plate might complement the previous analysis run and offer additional structural information on the bands identified as flavonoids, based on a potentially observable increase in peak intensity.

A limitation of this study is its qualitative nature. Therefore, future research should investigate the application of this method for quantitative analyses, similar to that established in the HPTLC-DPPH assay where bands responding to DPPH derivatization are quantified for their antioxidant activity as gallic acid equivalent using a corresponding gallic acid standard curve. An extensive study including accuracy, precision, and percent recovery studies using known amounts of various flavonoids as well as studies investigating the use of a suitable reference flavonoid (e.g., rutin) to quantify responses to derivatization with AlCl_3_ as rutin-equivalents needs to be performed to investigate the potential quantitative dimension of the proposed HPTLC-AlCl_3_ colorimetric method. Nonetheless, this study presents novel qualitative findings that will complement the traditional TFC determination and benefit the analysis of flavonoid-containing natural products as was demonstrated with the investigation of rutin- and naringin-containing supplements and also with the analysis of a red clover honey extract (Table 3). The methanolic solutions prepared from the rutin and naringin containing supplements showed a bathochromic shift, yielding absorbance maxima at 402 and 386 nm, respectively, after derivatization with AlCl_3_, thus complying with the typical pattern proposed as a positive identification for flavonoids using this method. On derivatization with NaNO_2_-AlCl_3_-NaOH, consistent with the patterns established in this study, the previously recorded λ_max_ values remained unchanged for both samples, but a 26.5% increase in absorbance intensity was noted for the rutin supplement only as this flavonoid alone contains a suitable substitution pattern in ring B that will allow the formation of a nitroxyl chelate. A red clover honey, reported to contain isoflavonoids [14], was also investigated to confirm the applicability of the optimized method (Table 3). The observed bathochromic shift of one of the bands recorded for this honey extract on derivatization with AlCl_3_ confirms its flavonoid characteristics. On derivatization with NaNO_2_-AlCl_3_-NaOH this peak maximum and its absorbance intensity remained unchanged, suggesting the lack of vicinal aromatic diols. Previous studies confirmed the presence of two isoflavones, genistein and daidzein, in red clover honey [15] which have distinct λ_max_ values of 370 and 307 nm (Table 1 and Figure 4f). Considering their substitution patterns on rings A, B, and C it can be anticipated that only one of them, genistein, will respond to AlCl_3_ derivatization with a bathochromic shift as daidzein is missing the necessary substitution pattern on rings A and C to facilitate Al^3+^ chelation. Without the presence of vicinal aromatic diols, it can also be assumed that genistein will not respond with an increase in absorbance intensity to treatment with NaNO_2_-AlCl_3_-NaOH. These anticipated observations were confirmed for the one band that could be identified in this study’s analysis of red clover extract. The band in question presented with an Rf value of 0.46 using a mixture of dichloromethane:ethyl acetate:acetic acid (12:1:12, *v*/*v*/*v*) as mobile phase, which is the same Rf value that was previously established for genistein using the identical mobile phase [14]. Using this chromatographic system, daidzein can be expected to present a band at around Rf 0.35, but given the lack of a substitution pattern suitable for chelation with Al^3+^, this band could not be identified in this study on derivatization with AlCl_3_. Daidzein is one of the few flavonoids that give a false negative response using the AlCl_3_ colorimetric method. This is, however, not a specific limitation of the HPTLC-AlCl_3_ method developed in this study, but a general limitation observed for this sub-class of flavonoids.

## 4. Materials and Methods

### 4.1. Chemicals and Reagents

All reagents and solvents used were of analytical grade. Naringin was obtained from Alfa Aesar (Morecambe, UK), all other flavonoids used in this study and gallic acid were sourced from ChemFaces (Wuhan, China), methanol was purchased from Scharlau (Barcelona, Spain), and ethyl acetate, glacial acetic acid, and formic acid from Ajax Finechem (Cheltenham, Australia). Anhydrous aluminium chloride, NaNO_2_, and NaOH were obtained from Sigma-Aldrich (Darmstadt, Germany). Figure 4 shows the chemical structures of all flavonoids used in the study alongside the non-flavonoid compounds that served as negative control.

For each of the investigated standards, the type and number of potential chelation sites upon reaction with either AlCl_3_ or NaNO_2_-AlCl_3_-NaOH were identified ((Table 1 and Tables S1–S6 in Supplementary Materials) based on their specific chemical structure (Figure 4) and the proposed chelate formation options (Figure 2).

### 4.2. Commercial Supplements and Clover Honey

Commercial rutin capsules (450 mg rutin per capsule) were purchased from Now Foods (Bloomingdale, IL, USA). Naringin capsules (500 mg naringin per capsule) were sourced from Swanson Health Products (Fargo, ND, USA). The red clover honey was produced at Shenton Park Field Station at the University of Western Australia.

### 4.3. Reagent and Sample Preparation

All flavonoid and non-flavonoid standards were prepared in a concentration of 0.5 mg/mL in methanol. Methanolic solutions of commercial rutin and naringin capsules were prepared at a concentration of 50 μg/mL. An organic honey extract was prepared by dissolving 1 g of red clover honey in 2 mL of deionized water, followed by three subsequent extractions with 5 mL of acetonitrile and dichloromethane (1:1, *v*/*v*). The combined organic extracts were dried with anhydrous MgSO_4_, filtered, and the solvent evaporated under a nitrogen stream before being reconstituted in 100 µL of methanol to prepare a honey extract solution for HPTLC analysis. A mixture of ethyl acetate:methanol:glacial acetic acid:formic acid (11:1:1:1, *v*/*v*/*v*/*v*) was prepared as a mobile phase for HPTLC analysis of all flavonoids, non-flavonoids, and commercial flavonoid-containing supplements, whereas dichloromethane:ethyl acetate:acetic acid (12:1:12, *v*/*v*/*v*) was used for clover honey extract in this study [14].

### 4.4. Method Development and Optimization

According to the literature, the total flavonoid colorimetric method is commonly carried out with methanolic solutions of aluminium chloride, sodium nitrite, and sodium hydroxide at concentrations of 10%, 5%, and 4%, respectively [17]. Based on this information, a comprehensive investigation was conducted as part of this study to determine the optimal concentration of these reagents for HPTLC derivatization by considering impacts of lower and also higher concentrations of each spraying reagent on observed peak intensities. Kinetic studies were also carried out by assessing the obtained spectral results at various time points (0, 30, 60, 120, and 180 min) after applying the derivatization reagent to determine whether the analysis benefitted from a prolonged derivatization to allow for maximum chelation. Moreover, the potential benefits of heating after derivatization as well as multiple applications of the spraying reagent were also considered during this optimization process (Table 4). Five different types of flavonoids, namely rutin, quercitrin, apigenin, naringenin, and taxifolin, as well as gallic acid and aspirin as non-flavonoid negative controls, were used during this method development and optimization process. Based on the various experiments, optimized conditions for derivatization either with AlCl_3_ or a combination of NaNO_2_-AlCl_3_-NaOH could be determined (Table 4).

Following the optimization of the derivatization methods the spectra of all 40 flavonoid standards, non-flavonoids serving as negative control, and investigated natural products (rutin and naringin supplements and red clover honey) were first recorded prior to derivatization using a TLC scanner. Then the HPTLC plates were derivatized with a single application of 2% AlCl_3_ (no heating, spectrum immediately recorded after spraying) as spray reagent 1 followed by another UV–Vis spectral analysis. In a separate analysis run, the spectra of all the analysed flavonoids, non-flavonoids, and natural product samples were also recorded after derivatization with a mixture of 2% NaNO_2_-AlCl_3_-NaOH (no heating spectrum recorded immediately after spraying) as spray reagent 2. The obtained results following derivatization with spraying reagents 1 and 2 were then compared with the respective compound spectra prior to derivatization to record any bathochromic shifts. The absorbance intensity upon derivatization with 2% AlCl_3_ and 2% NaNO_2_-AlCl_3_-NaOH was also recorded, and percentage changes determined (Table 1).

### 4.5. Instrumentation

The flavonoid standards and negative controls were applied at a volume of 4 µL as 8 mm bands at 10 mm from the lower edge of the HPTLC plate at a rate of 150 nLs^−1^ using a semiautomated HPTLC application device (Linomat 5, CAMAG, Muttenz, Switzerland). The application volume for the commercial rutin and naringin supplement solutions were 4 µL while the application volume for the red clover honey extract was 7 µL. The chromatographic separation was performed on silica gel 60 F_254_ HPTLC plates (glass plates 20 × 10 cm) in a saturated (33% relative humidity) automated development chamber (ADC2, CAMAG). The plates were pre-saturated with the mobile phase for 5 min, automatically developed to 70 mm at room temperature, and dried for 5 min.

The obtained chromatographic results were documented using an HPTLC imaging device (TLC Visualizer 2, CAMAG) under 254 nm. After derivatization with 2% AlCl_3_ (spray reagent 1) and 2% NaNO_2_-AlCl_3_-NaOH (spray reagent 2) using an HPTLC derivatizer (blue nozzle, level 4 and 3 mL), the images were visualized at R366 nm. The chromatographic images were digitally processed and analysed using specialized HPTLC software (visionCATS 3.1, CAMAG) which was also used to control the individual instrumentation modules. The scanning of individual bands was carried out using a TLC Scanner 4 in both UV–Vis mode (190–900 nm) before and after derivatization.

## 5. Conclusions

This study introduces an optimized approach for the qualitative identification of flavonoids present in a sample upon HPTLC analysis using either AlCl_3_ or NaNO_2_-AlCl_3_-NaOH as derivatization reagents. The proposed method can complement the traditional AlCl_3_ colorimetric method to determine TFC. The flavonoid–Al^3+^ complex produces a bathochromic shift that results in a new absorbance maximum around 400 nm, which is a unique characteristic that allows the differentiation of flavonoids (with the exception of some isoflavonoids) from other compounds. Additionally, a mixture of NaNO_2_-AlCl_3_-NaOH as spray reagent might be helpful to unveil information about the presence/absence of aromatic vicinal diols in the identified flavonoids and thus might assist in structure identifications.

## Figures and Tables

**Table 1:** Bathochromic shift and increased intensity in different types of flavonoids.

Flavonoids		λ_max_ of Non-Derivatized Flavonoid (nm)	Number of Chelation Options between Rings A and C^1^	Chelation Option with Vicinal OH Groups in Ring B^1^	λ_max_ After Complexation with Al^3+^ (nm)	% Change in Absorbance Intensity after Derivatization with NaNO_2_-AlCl_3_-NaOH
Subclass	Example					
Flavonols	Myricetin	374, 264, 205	2	Yes	**418**, 317, 270, 210	16
	Kaempferitrin	342, 268, 197	1	No	**388**, 342, 275, 208	Unchanged^2^
	Fisetin	324, 267, 204	1	Yes	**397**, 321, 267, 205	5%
Flavanolols	Taxifolin	293, 220, 201	2	Yes	**390**, 313, 223, 203	64
Flavan-3-ol	Epicatechin	330, 280, 204	0	Yes	**400**, 280, 206	150
	Catechin	380, 281, 204	0	Yes	**400**, 281, 205	140
Flavones	Apigenin	333, 272, 199	1	No	**389**, 298, 206	Unchanged^2^
	Chrysin	316, 271, 196	1	No	**380**, 324, 280, 218	Unchanged^2^
	Luteolin	348, 269, 204	1	Yes	**381**, 272, 210	11
Flavanones	Hesperetin	291, 224, 200	1	No	**380**, 305, 224, 201	Unchanged^2^
	Naringin	287, 228, 196	1	No	**385**, 225, 197	Unchanged^2^
Isoflavones	Sissotrin	303, 262, 200	1	No	**373**, 271, 201	Unchanged ^2^
	Genistein	303, 260, 196	1	No	**370**, 270, 201	Unchanged ^2^
	Daidzein	306, 250, 196	0	No	307, 250, 195	Not applicable

^1^ chemical structures are shown in Figure 4 and ^2^ defined as less than a 5% increase in absorbance intensity.

**Table 2:** Bathochromic shift and increased intensity in non-flavonoids.

Non-Flavonoids	λ_max_ of Non-Derivatized Flavonoid (nm)	λ_max_ After Complexation with Al^3+^ (nm)
Gallic Acid	275, 219	292, 226
Acetyl salicylic acid	277, 230, 196	282, 237, 202
Salicylic Acid	310, 265, 198	315, 278, 203
Rosmarinic Acid	329, 280, 230	350, 234, 277, 285

**Table 3:** Flavonoid identification in natural products.

Analysed Sample	Rf	λ_max_ (nm) Before Derivatization	λ_max_ (nm) After Derivatization	% Increased Absorbance Intensity after Derivatization with NaNO_2_-AlCl_3_-NaOH
Rutin capsule ^1^	0.21	364, 266, 204	402, 267, 208	26.5
Naringin capsule ^2^	0.32	287, 226, 197	386, 317, 196	Unchanged
Red clover honey ^2^	0.46	303, 260, 196	370, 271, 199	Unchanged
	0.35	306, 250, 196	306, 250, 195	Unchanged

^1^ mixture of ethyl acetate:methanol:glacial acetic acid:formic acid (11:1:1:1, *v*/*v*/*v*/*v*) as the mobile phase; ^2^ mixture of dichloromethane:ethyl acetate:acetic acid (12:1:12, *v*/*v*/*v*) as the mobile phase.

**Table 4:** Observations and conclusion based on investigation of different derivatization conditions.

Spray Reagent	Experimental Condition	Observation	Optimized Condition
AlCl_3_	Single application of 2% AlCl_3_, spectral monitoring over 180 min	Maximum absorbance intensity directly after application (0 min)	Single application of 2% AlCl_3_, no heating, spectrum to be recorded immediately after spraying
	Three successive applications of 2% AlCl_3_, spectral monitoring over 180 min	Unchanged or decreasing absorbance intensity with multiple sprays	
	Single application of 10% AlCl_3_, spectral monitoring over 180 min	Decreasing absorbance intensity compared to a single application of 2% AlCl_3_	
	Single application of 2% AlCl_3_, plate heated to 100 °C for 3 min, spectral monitoring over 180 min	Degradation of peak	
	Single application of 15% AlCl_3_, spectral monitoring over 180 min	Decreasing absorbance intensity compared to a single application of 2% AlCl_3_	
NaNO_2_ followed by AlCl_3_ and NaOH	2% NaNO_2,-_AlCl_3_-NaOH, single application of each reagent separately, spectral monitoring over 180 min	Maximum absorbance intensity directly after application (0 min), time-consuming process with three subsequent derivatization steps	Single application of a mixture of 2% NaNO_2_-AlCl_3_-NaOH, no heating, spectrum to be recorded immediately after spraying
	2% NaNO_2_-AlCl_3_-NaOH applied as mixture in a single application, spectral monitoring over 180 min	Maximum absorbance intensity directly after application (0 min)	
	2% NaNO_2_-AlCl_3_-NaOH applied as mixture, two successive applications, spectral monitoring over 180 min	Unchanged or decreasing absorbance intensity compared to a single application	
	5% NaNO_2_, 10% AlCl_3_ and 4% NaOH, single application of each reagent separately, spectral monitoring over 180 min	Decreasing absorbance intensity compared to 2% reagent concentration and time-consuming	
	2% NaNO_2_-AlCl_3_-NaOH applied as mixture in a single application, plate heated to 100 °C for 3 min, spectral monitoring over 180 min	Degradation of peak	
	10% NaNO_2,_ 15% AlCl_3_ and 10% NaOH, single application of each reagent separately, spectral monitoring over 180 min	Decreasing absorbance intensity compared to 2% reagent concentration and time-consuming	

## Data Availability

The original contributions presented in the study are included in the article, further inquiries can be directed to the corresponding author.

## Supplementary Materials

The following supporting information can be downloaded at: https://www.mdpi.com/article/10.3390/molecules29215161/s1, Table S1: Bathochromic Shift and Increased Intensity in Flavonol; Table S2: Bathochromic shift and Increased Intensity in Flavanolol; Table S3: Bathochromic Shift and Increased Intensity in Flavan-3-ol; Table S4: Bathochromic Shift and Increased Intensity in Flavones; Table S5: Bathochromic Shift and Increased Intensity in Flavanones; Table S6: Bathochromic Shift and Increased Intensity in Isoflavones.
