# Supplementary material for: Investigating Flavonoids by HPTLC Analysis Using Aluminium Chloride as Derivatization Reagent
Source: Molecules. 2024 Oct 31;29(21):5161. doi: 10.3390/molecules29215161 (PMC11547264; doi:10.3390/molecules29215161)
Supplement: Supplementary file 1 [file molecules-29-05161-s001.zip › molecules-3269753-supplementary.pdf]

Supplementary File

Investigating Flavonoids by HPTLC Analysis using Aluminium Chloride as Derivatization Reagent

Sharmin Sultana [1,2], Md Lokman Hossain [1,2], Tom Sostaric [1], Lee Yong Lim [1,2], Kevin J. Foster [3,4], Cornelia Locher [1,2]*

[1] Division of Pharmacy, School of Allied Health, University of Western Australia, Perth 6009, Australia; sharmin.sultana@research.uwa.edu.au (S.S.); mdlokman.hossain@uwa.edu.au (M.L.H.); tom.sostaric@uwa.edu.au (T.S.); lee.lim@uwa.edu.au (L.Y.L.)
[2] Institute for Pediatric Perioperative Excellence, The University of Western Australia, Perth, WA 6009, Australia
[3] School of Agriculture and Environment, University of Western Australia, Crawley 6009, Australia; kevin.foster@uwa.edu.au (K.J.F.)
[4] Department of Primary Industries and Regional Department, Perth 6000, Australia

* Correspondence: Cornelia Locher (C.L.); connie.locher@uwa.edu.au

Flavonols

The 11 investigated flavonols all presented peak maxima around 400 nm (388-418 nm) on derivatization with $AlCl_3$, translating into a bathochromic shift of approximately 55 nm (36-75 nm) from the non-derivatized spectrum (Table S1). On derivatization with $NaNO_2$-$AlCl_3$-NaOH, the previously recorded absorbance maximum remained unchanged, but an increase in absorbance intensity could be noted in those standards that featured the chelation option with vicinal OH groups in ring B and thus were able to form the flavonoid-nitroxyl chelate, while an unchanged absorbance intensity was found for those flavonols that offered no chelation option in ring B. The only standard that did not comply with this pattern was fisetin.

Table S1. Bathochromic Shift and Increased Intensity in Flavonol

Flavonol	λ_{max} of non-derivatized flavonoid (nm)	Number of Chelation Options between Rings A and C[1]	Chelation Option with vicinal OH groups in Ring B[1]	λ_{max} after complexation with Al^{3+} (nm)	% Change in absorbance intensity after derivatization with $NaNO_2$-$AlCl_3$-NaOH
Rutin	365, 265, 204	1	Yes	**401**, 272,209	26
Quercitrin	355, 265, 204	1	Yes	**396**, 272, 207	30
Isoquercetin	293, 227, 195	1	Yes	**393**, 315, 227, 199	26
Quercetin	375, 264, 201	2	Yes	**416**, 313, 269, 204	13
Kaempferitrin	342, 268, 197	1	No	**388**, 342, 275, 208	Unchanged[2]
Narcissoside	365, 260, 202	1	No	**399**, 268, 208	Unchanged[2]

Tiliroside	316, 272, 194	1	No	**391**, 306, 275, 231, 199	Unchanged[2]
Galangin	364, 269, 196	2	No	**403**, 332, 274, 201	Unchanged[2]
Kaempferol	370, 269, 198	2	No	**411**, 306, 199	Unchanged[2]
Myricetin	374, 264, 205	2	Yes	**418**, 317, 270, 210	16
Fisetin	324, 267, 204	1	Yes	**397**, 321, 267, 205	5

[1] chemical structures are shown in figure 4 and [2] defined as less than a 5% increase in absorbance intensity.

Flavanolol

Only one flavanolol was analyzed in this study which presented an absorbance maximum of 390 nm on derivatization with $AlCl_3$, translating into a bathochromic shift of 97 nm from the non-derivatized spectrum (Table S2). On derivatization with $NaNO_2$-$AlCl_3$-NaOH, the previously recorded absorbance maximum remained unchanged, but an increase in absorbance intensity could be noted as the compound has a chelation option involving vicinal OH groups in ring B and therefore can form the flavonoid-nitroxyl chelate.

Table S2. Bathochromic shift and increased intensity in flavanolol

**Flavanonol**	**λ_{max} of non-derivatized flavonoid (nm)**	**Number of Chelation Options between Rings A and C[1]**	**Chelation Option with vicinal OH groups in Ring B[1]**	**λ_{max} after complexation with Al^{3+} (nm)**	**% Change in absorbance intensity after derivatization with $NaNO_2$-$AlCl_3$-NaOH**
Taxifolin	293, 220, 201	2	Yes	**390**, 313, 223, 203	64

[1] chemical structures are shown in figure 4.

Flavan-3-ol

The three investigated flavan-3-ols presented a new absorbance maximum around 400 nm on derivatization with $AlCl_3$, translating into a bathochromic shift from the non-derivatized spectrum of around 55 nm (36-75 nm) (Table S3). On derivatization with $NaNO_2$-$AlCl_3$-NaOH, this absorbance maximum remained unchanged, but an increase in absorbance intensity could be noted in all analyzed flavan-3-ols as they all presented chelation options involving vicinal OH groups in ring B, transforming into the corresponding flavonoid-nitroxyl chelates.

Table S3. Bathochromic Shift and Increased Intensity in Flavan-3-ol

Flavan-3-ol	λ_{max} of non-derivatized flavonoid (nm)	Number of Chelation Options between Rings A and C[1]	Chelation Option with vicinal OH groups in Ring B[1]	λ_{max} after complexation with Al^{3+} (nm)	% Change in absorbance intensity after derivatization with $NaNO_2$-$AlCl_3$-NaOH
Epicatechin	330, 280, 204	0	Yes	**400**, 280, 206	150
Epigallocatechin	320, 274, 208	0	Yes	**400**, 275, 209	160
Catechin	380, 281, 204	0	Yes	**400**, 281, 205	140

[1] chemical structures are shown in figure 4.

Flavones

The 12 investigated flavones presented a new absorbance maximum around 385 nm (380-389 nm) on derivatization with $AlCl_3$, representing a bathochromic shift from the non-derivatized spectrum of around 45 nm (30-65 nm) (Table S4). On derivatization with $NaNO_2$-$AlCl_3$-NaOH, this absorbance maximum remained unchanged, but an increase in absorbance intensity could be noted in those flavones that offered additional chelation options involving vicinal OH groups in ring B to transform into the respective flavonoid-nitroxyl chelates.

Table S4. Bathochromic Shift and Increased Intensity in Flavones

Flavones	λ_{max} of non-derivatized flavonoid (nm)	Number of Chelation Options between Rings A and C[1]	Chelation Option with vicinal OH groups in Ring B[1]	λ_{max} after complexation with Al^{3+} (nm)	% Change in absorbance intensity after derivatization with $NaNO_2$-$AlCl_3$-NaOH
Apigenin	333, 272, 199	1	No	**389**, 298, 206	Unchanged[2]
Chrysin	316, 271, 196	1	No	**380**, 324, 280, 218	Unchanged[2]
Luteolin-7-O-glucoside	342, 270, 195	1	Yes	**380**, 275, 196	15
Luteolin-6-C-glucoside	350, 275, 213	1	Yes	**380**, 281, 213	9
Luteolin-7-O-glucoronide	348, 273, 202	1	Yes	**383**, 276, 208	10
Luteolin	348, 269, 204	1	Yes	**381**, 272, 210	11
Diosmetin	342, 266, 201	1	No	**382**, 273, 211	Unchanged[2]
Acaetin	331, 271, 196	1	No	**380**, 276, 298, 207	Unchanged[2]
Vitexin	337, 274, 218	1	No	**380**, 276, 300, 226	Unchanged[2]

Isovitexin	336, 277, 200	1	No	**382**, 282, 207	Unchanged[2]
Baicalin	333, 270, 200	1	No	**380**, 291, 207	Unchanged[2]
Cosmosiin	332, 276, 200	1	No	**380**, 296, 201	Unchanged[2]

[1] chemical structures are shown in figure 4 and [2] defined as less than a 5% increase in absorbance intensity.

Flavanones

The five investigated flavanones presented an absorbance maximum of around 380 nm (380-385 nm) on derivatization with $AlCl_3$ that resulted from a bathochromic shift from the non-derivatized spectrum of around 95 nm (89-103 nm) (Table S5). On derivatization with $NaNO_2$-$AlCl_3$-NaOH, the previously recorded absorbance maximum and absorbance intensity remained unchanged for all the analyzed flavanones given their lack of vicinal OH groups in ring B.

Table S5. Bathochromic shift and increased intensity in flavanones

**Flavanones**	**λ_{max} of non-derivatized flavonoid (nm)**	**Number of Chelation Options between Rings A and C[1]**	**Chelation Option with vicinal OH groups in Ring B[1]**	**λ_{max} after complexation with Al^{3+} (nm)**	**% Change in absorbance intensity after derivatization with $NaNO_2$-$AlCl_3$-NaOH**
Pinocembrin	293, 216, 196	1	No	**385**, 218, 196	Unchanged[2]
Naringenin	293, 227, 195	1	No	**382**, 302, 227, 196	Unchanged[2]
Hesperetin	291, 224, 200	1	No	**380**, 305, 224, 201	Unchanged[2]
Hesperidin	287, 220, 200	1	No	**380**, 224, 306, 203	Unchanged[2]
Naringin	287, 228, 196	1	No	**385**, 225, 197	Unchanged[2]

[1] chemical structures are shown in figure 4 and [2] defined as less than a 5% increase in absorbance intensity.

Isoflavones

Among the eight investigated isoflavones, four presented an absorbance maximum around 370 nm (370-373 nm) on derivatization with $AlCl_3$ following a bathochromic shift of around 70 nm (67-70 nm) from the non-derivatized spectrum (Table S6), however, on derivatization with $NaNO_2$-$AlCl_3$-NaOH their absorbance intensity did not further increase due to a lack of vicinal OH groups in ring B. Isoflavones like formononetin, ononin, daidzein and daidzin did not respond to derivatization with $AlCl_3$ as they do not feature the necessary substitution patterns in rings A and C to allow for complexation with the metal cation. These specific isoflavones will therefore not be accounted for in this analysis nor will they be captured in the traditional $AlCl_3$ colorimetric method to determine TFC.

Table S6. Bathochromic shift and increased intensity in isoflavones

Isoflavones	λ_{max} of non-derivatized flavonoid (nm)	Number of Chelation Options between Rings A and C[1]	Chelation Option with vicinal OH groups in Ring B[1]	λ_{max} after complexation with Al^{3+} (nm)	% Change in absorbance intensity after derivatization with $NaNO_2$-$AlCl_3$-NaOH
Sissotrin	303, 262, 200	1	No	**373**, 271, 201	Unchanged[2]
Biochanin A	303, 261, 196	1	No	**370**, 270, 199	Unchanged[2]
Genistein	303, 260, 196	1	No	**370**, 270, 201	Unchanged[2]
Genistin	303, 261, 197	1	No	**370**, 271, 202	Unchanged[2]
Ononin	306, 259, 201	0	No	304, 258, 199	Not applicable
Formononetin	306, 251, 196	0	No	307, 252, 199	Not applicable
Daidzin	306, 258, 196	0	No	305, 258, 199	Not applicable
Daidzein	306, 250, 196	0	No	307, 250, 195	Not applicable

[1] chemical structures are shown in figure 4 and [2] defined as less than a 5% increase in absorbance intensity.
